# Beer Consumption Increases Human Attractiveness to Malaria Mosquitoes

**DOI:** 10.1371/journal.pone.0009546

**Published:** 2010-03-04

**Authors:** Thierry Lefèvre, Louis-Clément Gouagna, Kounbobr Roch Dabiré, Eric Elguero, Didier Fontenille, François Renaud, Carlo Costantini, Frédéric Thomas

**Affiliations:** 1 Génétique et Evolution des Maladies Infectieuses, UMR CNRS/IRD 2724, Montpellier, France; 2 Caractérisation et Contrôle des Populations de Vecteurs, IRD/UR 016, Montpellier, France; 3 Institut de Recherche en Science de la Santé, Bobo-Dioulasso, Burkina Faso; 4 Laboratoire de Parasitologie et d'Entomologie Médicale, Centre Muraz, Bobo-Dioulasso, Burkina Faso; 5 Organisation de Coordination pour la lutte contre les Endémies en Afrique Centrale, Yaoundé, Cameroun; 6 Institut de Recherche en Biologie Végétale, Université de Montréal, Montréal, Canada; University of Exeter, United Kingdom

## Abstract

**Background:**

Malaria and alcohol consumption both represent major public health problems. Alcohol consumption is rising in developing countries and, as efforts to manage malaria are expanded, understanding the links between malaria and alcohol consumption becomes crucial. Our aim was to ascertain the effect of beer consumption on human attractiveness to malaria mosquitoes in semi field conditions in Burkina Faso.

**Methodology/Principal Findings:**

We used a Y tube-olfactometer designed to take advantage of the whole body odour (breath and skin emanations) as a stimulus to gauge human attractiveness to *Anopheles gambiae* (the primary African malaria vector) before and after volunteers consumed either beer (n = 25 volunteers and a total of 2500 mosquitoes tested) or water (n = 18 volunteers and a total of 1800 mosquitoes). Water consumption had no effect on human attractiveness to *An. gambiae* mosquitoes, but beer consumption increased volunteer attractiveness. Body odours of volunteers who consumed beer increased mosquito activation (proportion of mosquitoes engaging in take-off and up-wind flight) and orientation (proportion of mosquitoes flying towards volunteers' odours). The level of exhaled carbon dioxide and body temperature had no effect on human attractiveness to mosquitoes. Despite individual volunteer variation, beer consumption consistently increased attractiveness to mosquitoes.

**Conclusions/Significance:**

These results suggest that beer consumption is a risk factor for malaria and needs to be integrated into public health policies for the design of control measures.

## Introduction

Despite control efforts, malaria remains a leading cause of worldwide morbidity and mortality [1], [2]. The rate of contact between vertebrate hosts and mosquito *Anopheles* vectors has long been recognised as a crucial determinant of malaria transmission [3]–[5], and successful malaria control depends on understanding the interactions between mosquitoes and humans (e.g. [6]–[9]). Predictions of malaria transmission usually assume that all individuals are at equal risk from *Anopheles* vector bites. However, there is now strong evidence that humans vary in their attractiveness to malaria mosquitoes [10]–[16] and hence, that host-vector contact is far from random [17], [18]. Recent studies have highlighted the importance of heterogeneous biting in determining the prevalence of malaria infection [19], [20], with one example showing that 20% of individuals account for 80% of all infections among African children [19]. Therefore it becomes of great strategic importance to identify the cause of variation in human attractiveness, and to develop malaria control targeting those who are bitten the most [19], [20].

*Anopheles gambiae* Giles *sensu stricto* (henceforth *An. gambiae*) is the primary malaria vector in Africa [21]. The tremendous vectorial capacity of this species is mainly determined by its strong preference for feeding on humans [22]. Besides factors such as heat and moisture [23], females of *An. gambiae* locate and orientate toward their human host primarily through olfactory cues [24], [25]. Each person has a distinctive body odour that results from the emission of several hundred volatile organic compounds present in the breath and produced by the skin (gland secretions in interaction with resident skin bacteria) [26], [27]. Additional factors such as diet, general health condition, or reproductive status can also act upon this distinct odour signature and determine the odour profile of an individual [26], [28], [29]. Because of their strong effects on odours, these factors have been considered as causes of the observed variation in human attractiveness to malaria mosquitoes. For instance, pregnant women are twice as attractive to *Anopheles* vectors as their non-pregnant counterparts and are thus at a greater risk for malaria [30]–[32].

Despite potential consequences on exposure to malaria mosquitoes, there is a serious lack of empirical evidence describing how diet affects human attractiveness to disease vectors. While one study suggested that people are more attractive to laboratory bred *Aedes* mosquitoes after beer consumption [33], the effect of beer consumption on attractiveness to malaria mosquitoes from natural populations remains untested. As alcohol consumption is rising in most endemic malaria areas [34], it is becoming urgent to assess its effects on human attractiveness to malaria vectors.

By using an experimental setting designed to accommodate entire body odour and breath as stimuli, we investigated the human attractiveness to a natural population of *An. gambiae* before and after beer or water consumption in a malaria endemic area in south-western Burkina Faso (West Africa).

## Methods

### Ethics Statement

All participants were adult volunteers enrolled after the nature of the studies was explained and verbal informed consent was obtained. The research presents no more than minimal risk or harm to the participants and involves no procedure for which written consent is required. The experimenters explained the study to the volunteers verbally in the language they can understand, providing all pertinent information (purposes, procedures, benefits) and allowing the volunteers ample opportunity to ask questions. Following this verbal explanation, the volunteers were provided sufficient time to consider whether or not to participate in the research. A technical staff witness was present during the recruitment process. The ethics committee of Burkina Faso and the institutional research committee of the Centre Muraz approved the recruitment procedure and the protocol described in this study (protocol approval number 15-2008/CE-CM).

### Volunteers and Drinks

All volunteers were Burkinabe adult males aged between 20 and 43 years in good health and not using any medication. On the day of experiment the participants were asked not to smoke, drink alcohol or use deodorants. A total of 43 participants were randomly assigned to the beer (n = 25 volunteers) or the water groups (n = 18 volunteers).

The beverage used in this experiment is a local beer called *dolo* with low alcohol content (∼3%) and prepared from fermented dough of sorghum. *Dolo* is the most commonly consumed alcoholic beverage in Burkina Faso with 40% of the total sorghum grain production used for its preparation [35], [36]. Dolo is predominantly consumed by males during the evening at specific production sites called “cabarets”. For our experiment, we bought the *dolo* from two different production sites in the district of Dioulassoba (Bobo-Dioulasso) between one to two hours before the start of the experiment. Participants from the water group drank potable tap water from Bobo-Dioulasso.

### Mosquitoes

Experiments were conducted using the F_1_ progeny of field-collected gravid *An. gambiae* from villages of the Kou Valley, located 30 km north of Bobo-Dioulasso in south-western Burkina Faso [37]. In this area, the *An. gambiae* complex is composed almost exclusively of *An. gambiae* s.s. with the M molecular form predominating [37], [38]. The mosquitoes were reared at 25°C in the insectary of the Institut de Recherches en Sciences de la Santé (IRSS) in Bobo-Dioulasso [39]. Groups of 50 adult female mosquitoes (3 to 4-day old) without prior access to a blood meal were randomly collected from the rearing cages 6–8 h before the start of the experiments, and placed in paper cups covered by a gauze [39].

### Experimental Procedures

The attractiveness of each volunteer was tested twice: before (first trial) and 15 minutes after (second trial) the consumption of either one litre of *dolo* (the average amount ingested by consumers at a “cabaret”) or one litre of water. Following oral administration, alcohol is quickly absorbed from the gastrointestinal tract into the blood and metabolised [40]. Fifteen minutes is a sufficient interval for alcohol to be present in blood, breath, urine and sweat [40], [41].

**Figure 1:**
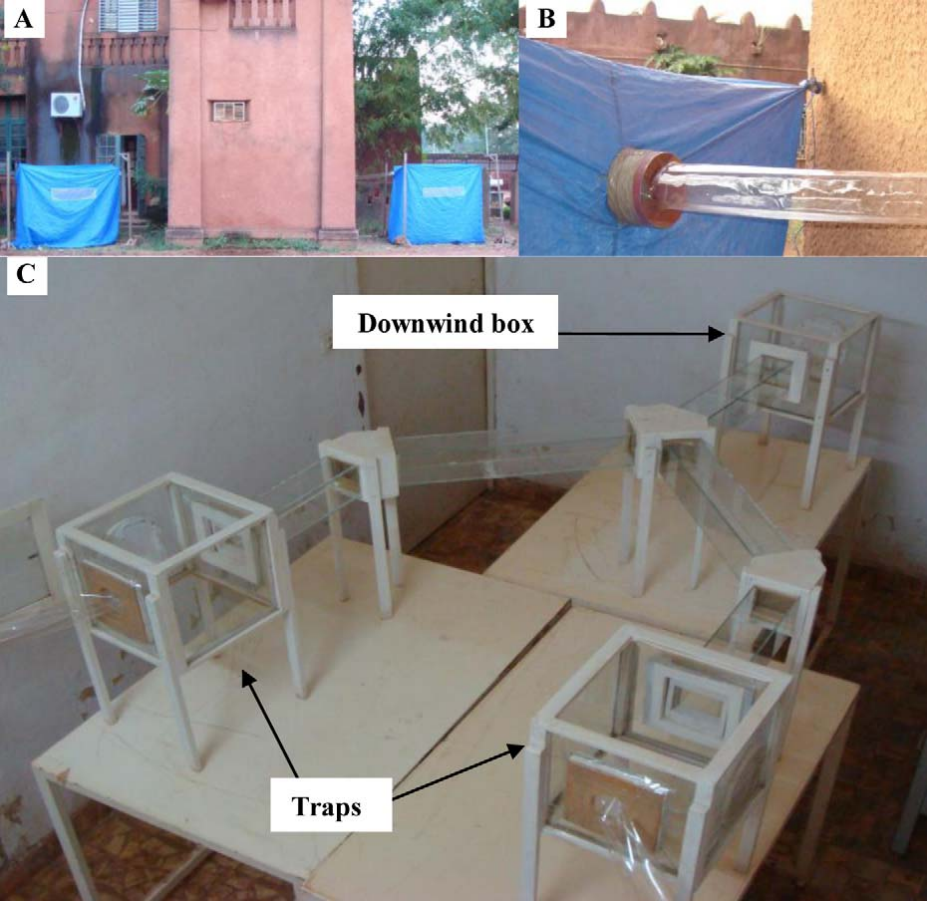
The bioassay. (A) The two tents set up outdoors and connected to the two traps of the Y-olfactometer by lay-flat tubing, and the olfactometer room located between the two tents. (B) Fan drawing air from a tent to the olfactometer via lay-flat tubing. (C) The Y tube-olfactometer.

The Y-olfactometer and the procedure are similar to those described previously [39]. Odours were directed from two polythene tents connected to the arms of a Y-tube olfactometer by polythene lay-flat tubing (figure 1). The tents were located outdoors and the olfactometer inside an experimental room (figure 1A). Fans drew air from the tents to the olfactometer, providing the odour laden air current against which mosquitoes were induced to fly (figure 1B). Gauze was placed at the junction of the lay-flat tubing with the traps to restrain responding mosquitoes inside the boxes and prevented them from flying into the tubing and into the tents (figure 1C). The air speed in the downwind arm of the Y-tube olfactometer was regulated at 20 cm/s using a 435-4 Testo multi-functional meter (Testo AG, Lenzkirch, Germany) equipped with a probe for degree of turbulence [range: 0 to +5 m/s, accuracy ± (0.03 m/s+4% of mv)] [39].

Batches of 50 mosquitoes were released into the downwind box of the Y-olfactometer (figure 1C) and given a choice between outdoor air and human odour. They were allowed to respond for 30 min. During this time frame, mosquitoes that responded to the stimuli left the downwind box and flew upwind into the traps from which they were retrieved (figure 1C). At the end of each test, the mosquitoes inside the two traps were removed with an aspirator and counted. The human odour consisted of one of four different treatments: Before Beer (BB), After Beer (AB), Before Water (BW), and After Water (AW) consumption. Human volunteers acting as odour sources sat shirtless on a chair inside the tent. The outdoor air treatment consisted of an empty tent with the four side walls open, so that outdoor air was drawn into the olfactometer [39]. Human odour and outdoor air stimuli were alternated between the right and left arm of the olfactometer to account for any side bias. All mosquitoes were tested only once. Experiments were carried out between 17:00 and 21:30. On each testing day 1–4 volunteers (randomly picked from the *dolo* and water groups) were tested. At the end of each trial, the carbon dioxide (CO_2_) concentration in the two arms of the Y-olfactometer was quantified using a 435-4 Testo multi-functional meter equipped with an indoor air quality probe [range: 0 to +10000 ppm CO2, accuracy: ±50 ppm CO2±2% of mv, 0 to +5000 ppm CO2] and an axillary measure of volunteer temperature was assessed. Finally, outdoor air was drawn in both arms of the Y-olfactometer to eliminate potential odour contaminants left from the previous trial. Every day, the olfactometer was washed with detergent and 70% alcohol. Latex gloves were worn by the experimenter to avoid contamination of the equipment. Experiments were conducted between September and October 2007.

### Statistics

Logistic regression by Generalized Linear Mixed Models (GLMM, binomial errors, logit link; analysed with the software R version 2.7.1 using the lme4 package) was used to investigate the effect of drink consumption (*dolo* and water) on volunteer attractiveness as characterised by two parameters:

- *Activation*, expressed as the proportion of mosquitoes caught in both traps out of the total number released in the downwind box; this is a measure of how many mosquitoes were activated by the odour stimuli, induced to take off and fly upwind into the traps [42].
- *Orientation*, expressed as the proportion of mosquitoes caught in the volunteer odour-baited trap out of the total number retrieved from both traps. This is a measure of the attractiveness of the volunteers' odours relative to the control outdoor air current.

The influence of several other explanatory variables were investigated by including these in the binomial models: position (whether volunteer odour was released from the left or right arm of the olfactometer), time of release, body temperature, mean CO_2_ concentration in the device on *activation*, and difference in CO**_2_** concentration between the traps on *orientation*.

The contribution of each explanatory term was tested sequentially, with non-significant terms removed from the model to produce the minimal model following standard stepwise deletion [43]. Only terms for which removal significantly (*P*<0.05) reduced the explanatory power of the model were retained in the minimal model [43]. All first-order interactions between significant variables were tested but none were significant.

Since the odours of volunteers were tested twice (both before and after drink consumption), the model was fitted by specifying drinks, CO2 concentration, position of treatment, time, and body temperature as fixed effects and the volunteer identity as a random effect [43].

To determine the consistency of volunteers' odours on mosquito behaviour we compared our variables (*activation*, *orientation*) before and after drinking with linear regression models. It was unclear whether an individual that induced high *activation* would also induce high *orientation*. We, therefore, also examined whether individuals' odours altered *activation* and *orientation* in tandem before drinking, after drinking *dolo*, or after drinking water with separate linear regressions. These analyses used untransformed proportions, since residuals were normally distributed and had homogeneic variance.

## Results

**Figure 2:**
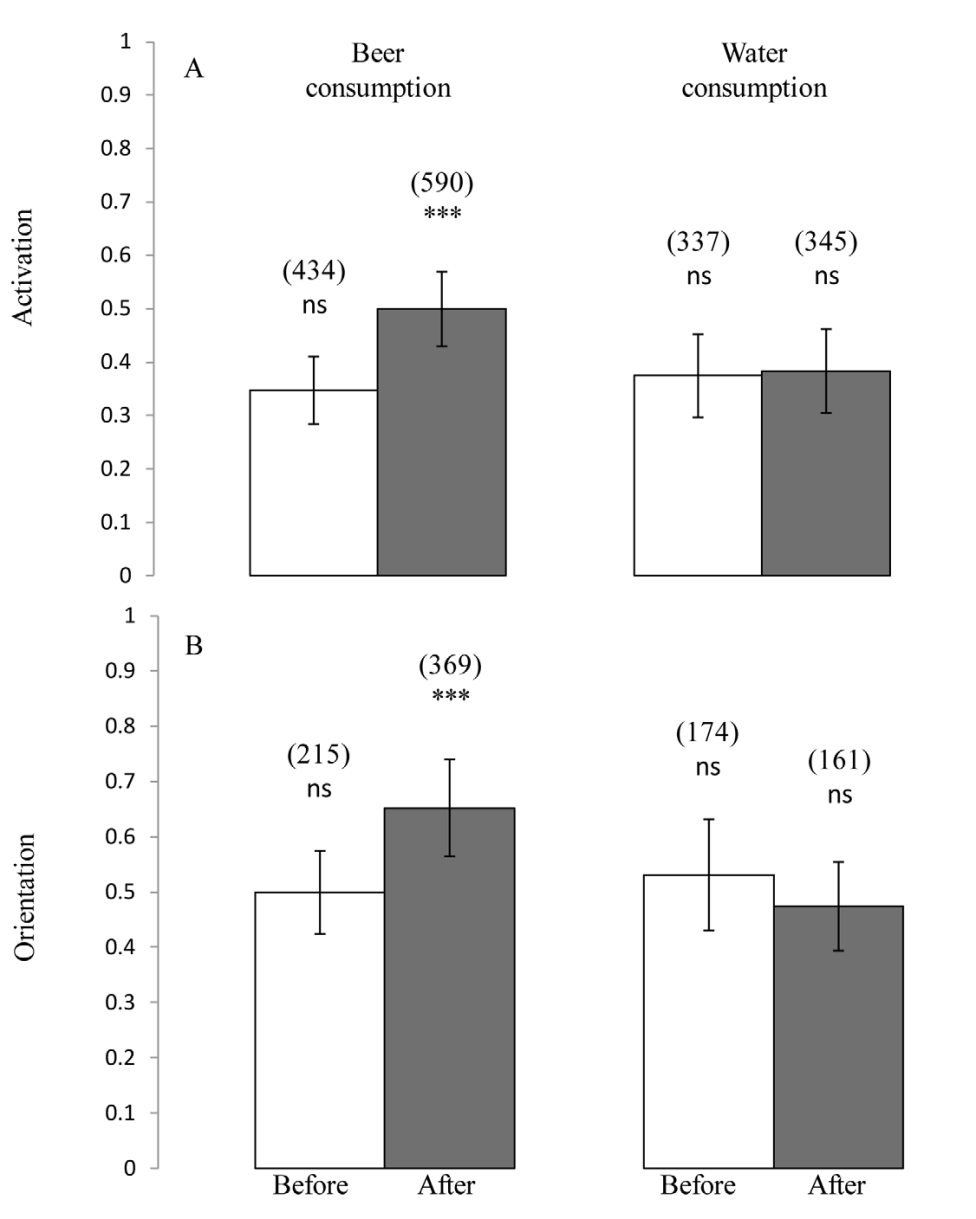
Beer consumption increases human attractiveness. (A) Effects of beer (n = 25 volunteers) or water (n = 18 volunteers) consumption on mosquito *activation*, expressed as the proportion of mosquitoes caught in both traps out of the total number released in the downwind box of the Y-olfactometer. In parentheses are the total numbers of mosquitoes entering both traps. (B) Effects of beer (n = 25 volunteers) or water (n = 18 volunteers) consumption on the mosquito *orientation*, expressed as the proportion of mosquitoes caught in the odour-baited trap out of the total number retrieved from both traps. In parentheses are indicated the numbers of mosquitoes entering the volunteer odour-baited trap. Error bars show 95% confidence interval of the mean proportion. Asterisks indicate significant effect of treatments on the response variables (GLMM); ns  =  not significant; ***  = P<0.001.

Beer consumption, as opposed to water consumption, significantly increased both the *activation* and *orientation* of *An. gambiae*. Beer consumption (‘After Beer’ (AB) treatment) activated significantly more of the mosquitoes (47%, GLMM; Odds Ratio (OR) = 1.63; 95% Confidence Interval (CI) = [1.41, 1.89]; P<0.001) than the three other treatments, ‘Before Beer’ (BB, 35%), ‘Before Water’ (BW, 37%) and ‘After Water’ (AW, 38%) (figure 2A). The proportion of activated mosquitoes retrieved from the volunteers' odour-baited trap was 50, 53 and 47% for the treatments BB, BW and AW respectively, indicating that mosquitoes did not orientate preferentially towards human odours relative to outdoor air (figure 2B). In contrast, 65% of the *An. gambiae* flying upwind, orientated toward the odour trap after the volunteers consumed beer, indicating a significant increase in *orientation* following beer consumption (OR = 1.77; CI = [1.36, 2.30]; *P*<0.001; figure 2B).

Axillary temperature decreased slightly after alcohol consumption (starting mean ± SE  = 36.3±0.09 C° to 36.1±0.08 C°; paired t-test, *t* = 3.2, df = 23, *P* = 0.004) and water consumption (from 36.3±0.11 C° to 36.2±0.11 C°; *t* = 2.2, df = 16, *P* = 0.045), but volunteer temperature did not affect mosquito *activation* (OR = 1.15; CI = [0.7, 1.5]; *P* = 0.7) or *orientation* (OR = 1.05; CI = [0.95, 1.15]; P = 0.6). The mean carbon dioxide CO_2_ concentration of outdoor air was 346±4 ppm. No significant difference in mean CO_2_ concentration exhaled by volunteers was found before and after beer (582±37 ppm and 568±38 ppm) or water consumptions (563±38 ppm and 589±42 ppm). The level of CO_2_ exhaled by volunteers in the Y-olfactometer had no effect on mosquito *activation* (OR = 1; CI = [0.99, 1.1]; *P* = 0.9), but higher levels of exhaled CO_2_ were associated with a lower degree of *orientation* (OR = 0.998; CI = [0.997, 0.999]; *P*<0.001). These findings indicate that the increased human attractiveness observed following beer consumption cannot be explained by increased carbon dioxide emission or body temperature.

The number of mosquitoes retrieved from the two traps increased over the evening, indicating an effect of release time on mosquito *activation* (OR = 1.25; CI = [1.14, 1.36]; *P*<0.001). Release time did not alter mosquito *orientation* (OR = 1.1; CI = [0.9, 1.32]; *P* = 0.3) and volunteer position (i.e. whether volunteer odour was released from the left or right arm of the olfactometer) did not affect mosquito *activation* (OR = 1.07; CI = [0.85, 1.29]; *P* = 0.57) or *orientation* (OR = 1.05; CI = [0.66, 1.44]; *P* = 0.8).

**Figure 3:**
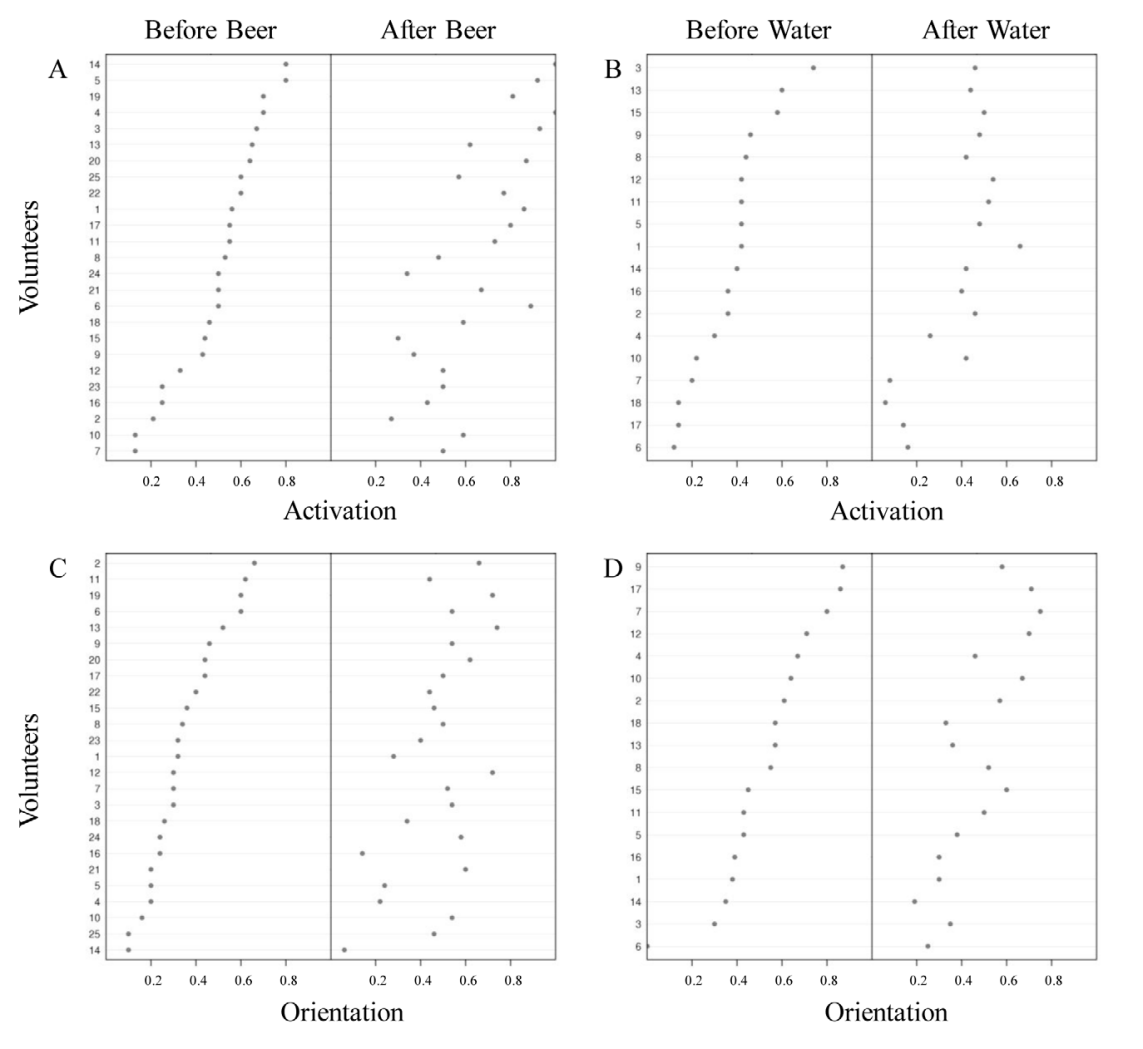
Variation in human attractiveness. (A) *Activation* scores for each volunteer before and after dolo consumption (n = 25 volunteers). (B) *Activation* scores for each volunteer before and after water consumption (n = 18 volunteers). (C) *Orientation* scores for each volunteer before and after dolo consumption (n = 25 volunteers). (D) *Orientation* scores for each volunteer before and after water consumption (n = 18 volunteers). For each panel, the volunteers are ranked from bottom (lowest score before drink consumption) to top (highest score before drink consumption).

*Activation* and *orientation* varied with the volunteer tested (figure 3). There was no relationship between *activation* and *orientation* (before drink consumption: *r^2^* = −0.016, *P* = 0.56; and after *dolo* consumption: *r^2^* = 0.07, *P* = 0.11; or after water consumption: *r^2^* = −0.05, *P* = 0.7). Volunteers that induced high mosquito *activation* did not systematically induce high mosquito *orientation*.

**Figure 4:**
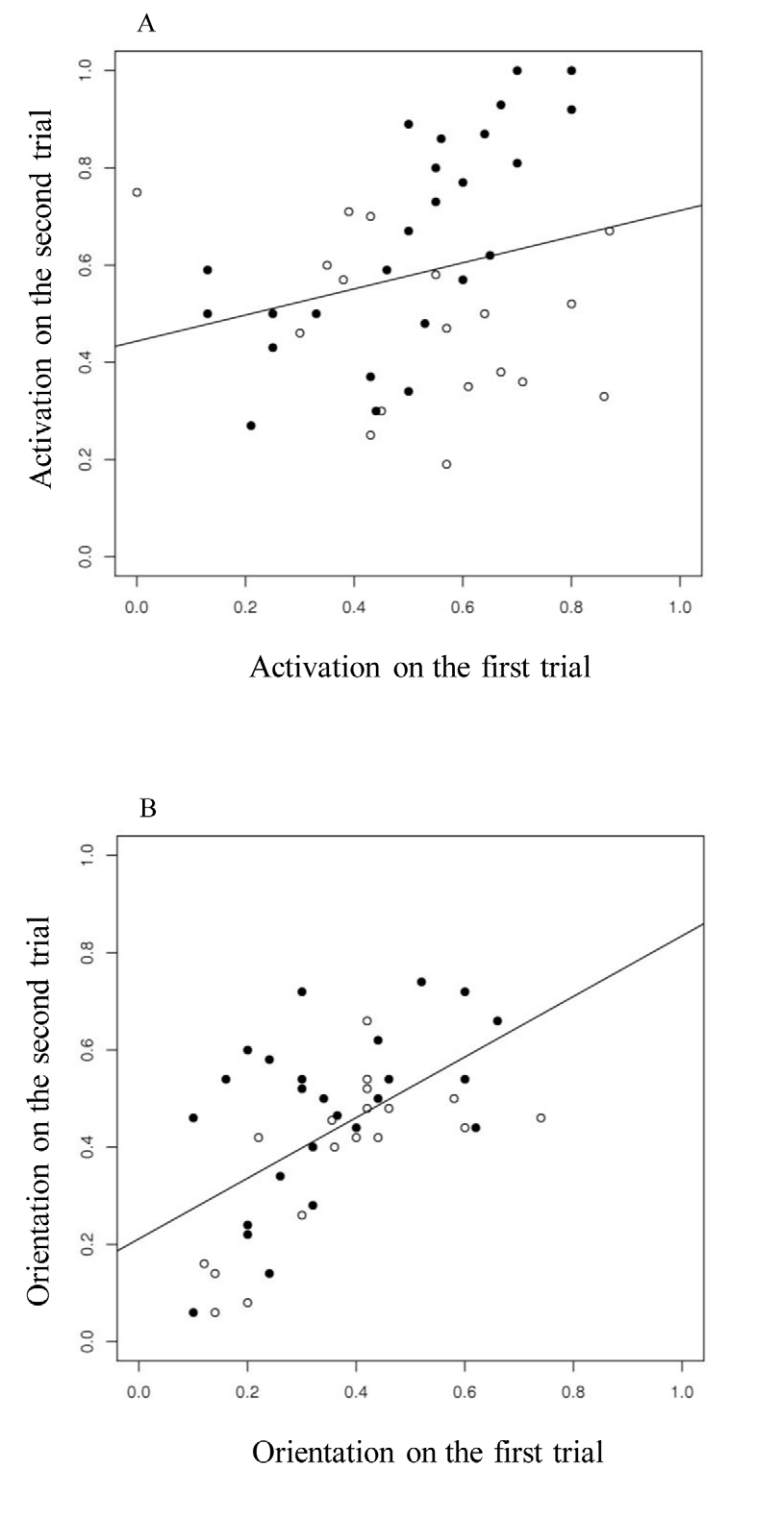
Consistencies in human attractiveness over the first and second trial. (A) Relationship between mosquito *activation* on the first and second trial. (B) Relationship between mosquito *orientation* on the first and second trial. Volunteers from the dolo group (n = 25) are represented by closed circles and those from the water group (n = 18) by open circles. The lines are the least squares regression lines.

Individual volunteers' odours affected mosquito behaviour consistently across trials (*activation: r^2^* = 0.31, *P*<0.001, figure 4A; *orientation: r^2^* = 0.37, *P*<0.001, figure 4B). Volunteers that induced high *activation* or *orientation* before drinking also induced high *activation* or *orientation* after drinking.

Overall, our findings indicate that, despite the individual differences of volunteers, beer consumption consistently increased volunteers' attractiveness to mosquitoes.

## Discussion

We explored the effect of beer consumption on human attractiveness to a natural population of *An. gambiae* using a Y-olfactometer designed to accommodate total body emanations as a source of odour stimuli. We found that beer consumption not only enhanced the number of mosquitoes that engage in odour-mediated upwind flight (mosquito *activation*) but also enhanced the strength of their odour-mediated anemotactic response (mosquito *orientation*). Mosquito *activation* and *orientation* are important parts of the natural host-seeking process of *An. gambiae* and any increases in these behaviours will facilitate vector-human contacts [23], [24]. Water consumption did not affect these mosquito behavioural responses, demonstrating that beer was solely responsible for increased human attractiveness. To the best of our knowledge, this study provides the first evidence that beer consumption increases human attractiveness to *An. gambiae*, which is the principal vector of malaria in Africa.

The proximate reasons for why people are more attractive after beer consumption are currently unclear. The increased attractiveness of pregnant women has been attributed to increased body temperatures and exhaled breath [30]. Here, higher body temperatures were not associated with higher attractiveness and beer consumption actually resulted in decreased body temperatures. Our results also indicate that increased exhaled breath cannot account for the observed increase in human attractiveness following beer consumption. Higher levels of exhaled breath (as measured with a CO_2_ analyser) were not associated with higher attractiveness and beer consumption did not affect CO_2_ expiration rate. We postulate that the metabolism of alcohol following beer consumption induces changes in breath and odour markers (i.e. increases the production of kairomones such as 1-octen-3-ol) that increases attractiveness to *An. gambiae*. Beyond this coincidental side effect of beer consumption, mosquitoes may have evolved preferences for people who recently consumed beer - possibly due to reduced host defensive behaviours or highly nutritious blood-meals. This hypothesis is appealing but requires further investigations.

Although beer consumption significantly increased the volunteer attractiveness relative to the outdoor air control (mosquito *orientation*), the absence of orientational bias toward volunteer odours in the three other treatments (BB, BW and AW) is intriguing. Similar results have been recently obtained using the same methodology and mosquito population [39]. In field situations, odour-mediated anemotaxis is an effective strategy to locate a vertebrate. Upon arrival in the vicinity of the host, additional cues such as warm, moist convective currents and host movement are exploited by the insect to orientate toward the host. Therefore, the absence of orientational bias for the volunteer odour-baited trap may have stemmed from the fact that our bioassay removes some of these host-related short-range stimuli.

Unsurprisingly *An. gambiae activation* increased over the evening (17:00 to 21:30). Like most anopheline species, *An. gambiae* is a night biter. In this species, the biting cycle starts at sunset and rises to peak between 24:00 and 1:00 [24]. Thus, the positive relationship between mosquito *activation* and time simply reflects the natural circadian flight activity, the process by which hungry females of *An. gambiae* engage in non-oriented flight to optimise their chance of encountering host stimuli [44]. Upon contact with odour stimuli, An. gambiae then switches from this appetitive search to the actual host location behaviours.

We found that higher levels of expired CO_2_ were associated with lower mosquito *orientation*. This result is in apparent contradiction with the general acceptance that blood-feeding insects are attracted to carbon dioxide. Despite thorough investigations, the role of CO_2_ in host-finding by *An. gambiae* mosquitoes remains equivocal [24]. At least three lines of arguments can be advanced to resolve this apparent contradiction. First, it is increasingly recognized that while CO_2_, a compound exhaled by all mammals, induces mosquito *activation*, it does not provide accurate orientational cues to the anthropophilic *An. gambiae* [24], [42]. Second, compounds which are termed attractants can also act as repellents at high concentrations [45]. In natural conditions, human exhalations are dispersed and strongly diluted before contacting host-seeking mosquitoes [42]. Accordingly, the CO_2_ concentration released in the volunteer trap (575 ppm on average) may have been too strong, resulting in a negative effect on mosquito *orientation* [15]. Finally, the fine-scale structure of the CO_2_ plume (continuous vs. pulsed stream) is known to affect the *orientation*, with a continuous plume reducing the behavioural responses of mosquitoes [24], [46]. We do not know the structure of the CO_2_ plume in this bioassay.

Alcohol consumption is a widespread phenomenon throughout the world and represents one of the most pressing global health priorities [47]. The alcoholic beverage used in this experiment is a very popular drink in West Africa [36]. Therefore, the increased attractiveness following beer consumption found here raises crucial issues regarding strategic planning for malaria control. Recent models have stressed that local malaria control can only be reached if people who are bitten the most can be identified [20]. By ascertaining beer consumption as a risk factor, our study has identified a potential underlying cause of heterogeneous biting, and hence provides insights into the feasibility of targeted interventions.

The outlook may be even worse if we consider that alcohol contributes substantially to the global burden of diseases [48], especially by compromising the host immune defence against parasites. Numerous studies have demonstrated that moderate and chronic alcohol consumptions can have strong immunosuppressive effects [49]. Therefore, people who drink beer are not only at higher risk of exposure to malaria mosquitoes but could also be more vulnerable to the *Plasmodium* parasites. Given the importance of beer consumption in the populations that are most at risk from malaria, this is a possibility that requires attention.

To eliminate the possibility that other active ingredients in beer apart from alcohol could be driving the observed effects, future studies are needed to test whether consumption of other alcoholic beverages also increases the risk of being bitten by *An. gambiae*, and hence being infected with malaria parasites in natural situations. Finally, it is crucial to investigate the effect of alcohol consumption on the success of gametocyte (the *Plasmodium* infective stage for mosquitoes) production. Expanding experiments and observations on the attractiveness of people consuming alcohol to malaria mosquitoes and *Plasmodium sp.* development in these hosts should allow us to gauge the role of alcohol consumption on the transmission dynamic of malaria.
